# Workforce wellbeing centres and their positive role for wellbeing and presenteeism in healthcare workers during the COVID-19 pandemic: secondary analysis of COVID-Well data

**DOI:** 10.1186/s12913-024-10730-9

**Published:** 2024-03-06

**Authors:** Holly Blake, Helen Mancini, Emma Coyne, Joanne Cooper, Natalia Stanulewicz-Buckley

**Affiliations:** 1 School of Health Sciences, University of Nottingham, Nottingham, UK; 2 NIHR Nottingham Biomedical Research Centre, Nottingham, UK; 3 Human Resources, Nottingham University Hospitals NHS Trust, Nottingham, UK; 4 Clinical Psychology Department, Nottingham University Hospitals NHS Trust, Nottingham, UK; 5 Nursing and Midwifery, Nottingham University Hospitals NHS Trust, Nottingham, UK; 6 School of Psychology, Aston University, Birmingham, UK

**Keywords:** Workforce, Wellbeing, Presenteeism, Healthcare workers, COVID-19, Pandemic

## Abstract

**Background:**

Supported wellbeing centres established during the COVID-19 pandemic provided high quality rest spaces and access to peer-to-peer psychological first aid for healthcare workers (HCWs). The centres were well accessed and valued by HCWs, but their relationship with wellbeing and job-related factors is not well established. The aim of this study was to explore the relationship between wellbeing centre use, HCWs wellbeing and job-related factors (job stressfulness, job satisfaction, presenteeism, turnover intentions).

**Methods:**

Secondary analysis of data from 819 HCWs from an acute hospital trust who completed an online survey in April-July 2020, as part of the COVID-Well study. Measures included the Warwick Edinburgh Mental Wellbeing Scale, and four single-item global measures of job stressfulness, job satisfaction, presenteeism and turnover intentions. ANCOVA models and regression analyses were conducted on these data.

**Results:**

HCWs who had not accessed the wellbeing centres had lower wellbeing (β = 0.12, *p* < .001), higher job stressfulness (β = − 0.22, *p* < .001), lower job satisfaction (β = 0.39, *p* < .001), higher presenteeism (β = − 0.22, *p* < .001) and were of younger age (β = 0.09, *p* = .002). Centre use was associated with wellbeing irrespective of job stressfulness. Those reporting presenteeism and who accessed the centre (M = 3.30, SE = 0.04) had higher wellbeing than those who accessed the centre but did not report presenteeism (M = 3.06, SE = 0.04) (F(1, 791) = 18.65, *p* < .001, η_p_^2^ = 0.02). Centre use was not significantly associated with turnover intentions (B = − 0.30, *p* = .13; Wald = 2.26; odds = 0.74), while job stress and job satisfaction showed significant effects.

**Conclusions:**

Accessing wellbeing centres was associated with higher wellbeing of HCWs, particularly for those reporting presenteeism. Therefore, the centres may have provided greatest respite and restoration for those present at work but not in optimal health. Younger workers were disproportionately affected in terms of wellbeing, and targeted support for this population is needed. Strategies to decrease presenteeism and maximise job satisfaction are urgently required. Healthcare organisations should provide rest spaces and psychological support to HCWs for the long-term, as part of a systems-wide approach to improving workforce health and wellbeing.

**Supplementary Information:**

The online version contains supplementary material available at 10.1186/s12913-024-10730-9.

## Background

The coronavirus (COVID-19) pandemic negatively impacted the mental wellbeing of healthcare workers (HCWs), globally [1–10]. Low wellbeing in healthcare workers has implications for patient safety [11, 12] and predicts turnover intentions [13, 14]. Studies have identified a negative relationship between wellbeing and job stress [15, 16], as well as a negative relationship between wellbeing and presenteeism [17–19], and a positive relationship between wellbeing and job satisfaction in HCWs [20–22]. Nonetheless, a more complex analysis of the interactions between those constructs is needed.

Systematic reviews conducted prior to the pandemic discuss various interventions that improve health and mental wellbeing in HCWs [23, 24]. However, there is a lack of published evidence reporting on interventions aimed at improving the mental health and wellbeing of HCWs during the COVID-19 pandemic [25, 26]. While a Cochrane review [26] identified 16 studies that reported implementation of an intervention aimed at supporting the mental health of frontline workers during disease outbreaks, only four had been implemented during COVID-19 pandemic. Moreover, only one of these studies was conducted in the United Kingdom (UK); a digital psychological support package developed within three weeks of pandemic outbreak [27]. This had global reach and impact [28] but represents only one, remotely delivered, approach to wellbeing support.

Subsequently, the COVID-Well studies [1, 29] were the first to report on the implementation and evaluation of COVID-19 supported wellbeing centres for HCWs in an acute hospital setting. Two wellbeing centres were established at two sites of an acute hospital trust in the UK. The centres provided high-quality rest spaces and were staffed by 134 ‘wellbeing buddies’ (trained in Psychological First Aid: PFA) providing face-to-face, peer-to-peer support to visitors, hence named ‘supported’ centres. Access to psychological support (e.g., PFA), regular work breaks and spaces for rest and reflection have been strongly advocated in the UK in recent years [30–32]. In line with this, PFA was used to provide emotional support to HCWs during the COVID-19 pandemic [33–35]. The World Health Organization [36] developed PFA, which focuses on active listening, the provision of practical care and signposting to further support. PFA training can improve basic psychological skills in frontline workers [37], and is advocated for those working in high-risk environments, such as the healthcare setting [38]. Work breaks are recognised as key to fostering a caring environment by preventing stress, burnout, and compassion fatigue [39], and the provision of high-quality rest spaces has been shown to impact on staff morale, well-being, and quality of patient care [1, 40].

The COVID-Well study [29] showed that these COVID-19 staff wellbeing centres were highly accessed during the first pandemic surge in the UK (14,934 facility visits over 17 weeks). Qualitative interviews with HCWs and wellbeing buddies revealed positive views towards this provision and broad benefits for workforce wellbeing, teamwork, and care quality [1]. These prior studies described the wellbeing and characteristics of those who did, and did not visit the wellbeing centres, and explored the views of HCWs and service providers towards the intervention. However, these studies did not explore the relationship between centre access, HCWs wellbeing and job-related factors.

The aim of this study was to expand on those previous findings, and to quantitatively explore the relationship between wellbeing centre use, HCWs wellbeing, and job-related factors (job stressfulness, job satisfaction, presenteeism, and turnover intentions). A better understanding of this relationship may help to inform approaches to the improvement of HCWs wellbeing and reducing turnover intentions in the healthcare workforce, particularly within the context of high stress working conditions and emergency situations (such as a pandemic). This has not been examined in our previous publications [1, 29]. To address this aim, the research questions in the current paper were: (1) Are job-related factors in the context of pandemic (i.e., job stress, job satisfaction, presenteeism, wellbeing centre use) associated with wellbeing in HCWs? (2) What is the relationship between job stress and centre use and its role for wellbeing scores? (3) What is the relationship between job satisfaction and centre use and its role for wellbeing scores? (4) Does wellbeing centre access explain turnover intentions?

In line with previous literature examining the relationships between job-related factors and wellbeing (e.g. [41, 42]), it was expected that job stress would show a negative association with HCWs wellbeing, while job satisfaction (e.g. [43–45]) would have a positive association with wellbeing. The novel aspect that this study adds, however, is the examination of a potential moderating effect of wellbeing centre use. Here, it was expected that wellbeing centre use would strengthen the link between wellbeing and job satisfaction but weaken the link between wellbeing and job stress. Lastly, it was explored whether wellbeing centre use would decrease turnover intentions. However, as turnover intentions is relatively complex and impacted by a multitude of factors (e.g., job satisfaction, but also organisational/job commitment [46–48], job ambiguity, participation in decision making, etc. [49]). it was predicted that any negative association between wellbeing centre use and turnover intentions would be relatively small, if observed at all.

## Methods

### Study design

Cross-sectional data from the COVID-Well study [29] were re-analysed, to specifically explore the relationships between wellbeing centre use, job-related factors (i.e., job stress, job satisfaction, presenteeism) and HCWs wellbeing, as well as turnover intentions. Such analyses were not performed in the previous COVID-Well publications [1, 29].

### Setting and participants

The setting was an acute hospital trust in the UK, with two COVID-19 staff wellbeing centres that had been established on different hospital sites in April 2020. Eligible participants were HCWs from the same hospital trust (HCWs is used here to refer to paid employees, bank staff and contracted volunteers from any occupational group). The study included 806 HCW participants who completed a questionnaire survey.

### Procedures

Data were collected using a web-based survey hosted on JISC Online Surveys (https://www.onlinesurveys.ac.uk), that was open for six weeks between July – August 2020 and was promoted via employee mailing lists and social media. Potential participants were signposted to an online participant information sheet containing a link to an online survey. The information sheet indicated that participants could choose whether or not to take part, and that they were providing informed consent to participate by submitting their responses. Data were collected immediately after the first surge of COVID-19 in the UK and following 17 weeks availability of supported wellbeing centres to HCWs. The study was carried out in accordance with the Helsinki Declaration. The protocol was reviewed and approved by University of Nottingham Faculty of Medicine and Health Sciences Research Ethics Committee (Ref. 16–0520) and the local NHS R&D department (Ref. 20-269 C). The study procedures and intervention are fully described elsewhere [29]. Here, a brief description is provided in Table 1, which was created in line with the information provided in previous publications (ibidem).

### Intervention

The intervention is summarised in line with the TIDieR checklist for intervention description and replication [50] (Table 1). The intervention was delivered in accordance with the British Psychological Society Code of Ethics and Conduct.

**Table 1:** Intervention description for COVID-19 supported wellbeing centres

TIDieR checklist item	Study detail
BRIEF NAME: Provide the name or a phrase that describes the intervention.	COVID-Well: Supported Wellbeing Centres
WHY: Describe any rationale, theory, or goal of the elements essential to the intervention.	Provision of high-quality rest spaces for HCWs will improve wellbeing through providing work breaks, rest, respite, and opportunity for social contact. Providing access to psychological first aid within the centres will improve wellbeing and reduce presenteeism through providing point-of-care support and signposting for the prevention or management of psychological crises in HCWs during the pandemic.
WHAT: *Materials*: Describe any physical or informational materials used in the intervention, including those provided to participants or used in intervention delivery or in training of intervention providers. Provide information on where the materials can be accessed (e.g., online appendix, URL). *Procedures*: Describe each of the procedures, activities, and/or processes used in the intervention, including any enabling or support activities.	Centres were designed to be relaxing spaces, with refreshments, comfortable seating, relaxing music, low-level lighting, plants, and an aromatherapy pod. Charitable donations for employees (i.e., personal care packages, wash bags, toiletries, snacks, and washable uniform bags) were available for a limited time only. PFA (active listening, social support, signposting) was provided by trained wellbeing support workers called ‘wellbeing buddies’. There were two buddies per site during opening hours. Dedicated partitioned areas within the centres provided privacy and space for buddies to deliver emotional support and signposting (e.g., to GPs, counselling and other services, telephone crisis hotlines, COVID-19 testing, self-care resources). Buddies were responsible for ensuring adherence to health and safety regulations within the facilities, including social distancing guidelines.
WHO PROVIDED: For each category of intervention provider (e.g., psychologist, nursing assistant), describe their expertise, background and any specific training given.	One hundred and thirty-four wellbeing buddies opted into the role and were trained in PFA by NHS clinical psychologists, who also provided the buddies with regular supervision and drop-in sessions to address their queries, provide mentoring and psychological support. Some, but not all, of the buddies had prior experience in counselling or patient-facing roles that involved ‘active listening’, although there were no pre-requisites for this role as all volunteers received training and support. Buddies were NHS employees who had reduced workload in their main roles during the pandemic due to temporary closures of clinics or services. The minimum time commitment for any buddy was a single 4-h shift and the level of time commitment varied with some buddies completing 1–2 shifts in total, and others completing several shifts per week. However, all buddies continued to be employed in their main job while taking time out of this role to volunteer as a wellbeing buddy in the centres. Towards the end of the study period, buddies who had worked any shifts in the wellbeing centres during the pandemic were required to return fully to their usual roles.
HOW: Describe the modes of delivery (e.g., face-to-face or by some other mechanism, such as internet or telephone) of the intervention and whether it was provided individually or in a group.	The centres were accessed in person, PFA was provided face-to-face. Mode of delivery of the contact between wellbeing buddies and HCWs was at HCWs preference (i.e., contact could be individual, or small group). Signposting included remote support (i.e., web-based materials, digital apps, telephone support (employee assistance programme)).
WHERE: Describe the type(s) of location(s) where the intervention occurred, including any necessary infrastructure or relevant features.	Two wellbeing centres, located at different hospital sites of the same NHS trust. Both centres had comparable facilities, although one (A) was a purpose-built wellbeing room, and the other (B) was a converted hospital ward that had previously been used for training.
WHEN and HOW MUCH: Describe the number of times the intervention was delivered and over what period of time including the number of sessions, their schedule, and their duration, intensity or dose.	The centres were opened on 06 April 2020 and could be accessed by employees between 08:00 and 20:00 on seven days of the week. The dose and frequency of intervention was determined by HCWs’ personal preference and/or break schedule.
TAILORING: If the intervention was planned to be personalised, titrated, or adapted, then describe what, why, when, and how.	Centre visitors could utilise the facilities according to their personal preference. This could be quiet time-out and personal space (e.g., for rest, reflection, to read, to rehydrate), social contact (e.g., with colleagues/peers, or wellbeing buddies) or emotional support (e.g., PFA).
MODIFICATIONS: If the intervention was modified during the course of the study, describe the changes (what, why, when, and how).	Transition of buddies to prior job roles, coupled with analysis of usage data, informed a decision to change the centre opening hours to Monday–Friday 10:00–16:00 from week 9. Minor modification to planned centre facilities - charitable donations for employees (e.g., personal care packages, wash bags, toiletries, snacks, and washable uniform bags) were only available in the first few weeks, then moved to another location to manage volume and flow of visitors to centres and retain the primary purpose of the centres as a rest area. Both minor adjustments were made during intervention delivery period but prior to survey data collection.
HOW WELL: *Planned*: If intervention adherence or fidelity was assessed, describe how and by whom, and if any strategies were used to maintain or improve fidelity, describe them. *Actual*: If intervention adherence or fidelity was assessed, describe the extent to which the intervention was delivered as planned.	17-week service monitoring was undertaken. 14,934 facility visits were recorded across two sites (peak attendance in single week *n* = 2605). Facilities were highly valued, but the service model was resource intensive with 134 wellbeing buddies supporting the centres in pairs. Further detail on uptake, costs, delivery, and nature of wellbeing support provided is available in Blake and colleagues [1, 29].

PFA: Psychological first aid; NHS: National Health Service; Social distancing: at the time of the study the government recommendation was to maintain a 2-metre distance between people, where possible

Note: This table is adapted from text reported in previous publications [1, 29]

### Survey measures

Wellbeing was measured using the Warwick Edinburgh Mental Wellbeing Scale (WEMWBS) [51, 52]. The WEMWBS is a 14-item scale used to measure mental wellbeing in the general population. Responses are on a 1 to 5 Likert scale, with responses summed to generate a total score ranging from 14 to 70, where higher scores indicate more positive wellbeing (mean scores were used in the current analyses). WEMWBS has good psychometric properties demonstrating high content and criterion validity, internal consistency (Cronbach’s alpha 0.89–0.91) and high test-retest reliability (intra-class correlation coefficient 0.83) [52].

Other measures included four single-item global measures of job stressfulness [53], job satisfaction [54], turnover intentions [55] and presenteeism [56]. Job stressfulness was measured by the item: ‘In general, how stressful do you find your job?’ with responses on a 5-point scale ranging from 1 = ‘not at all stressful’ through to 5 = ‘extremely stressful’. Job satisfaction was measured by the item: ‘Taking everything into consideration, how do you feel about your job as a whole?’ with responses ranging from 1 = extremely dissatisfied through to 5 = extremely satisfied. Turnover intentions were assessed using the item: ‘Are you considering leaving your job?’ (yes or no). Presenteeism was assessed using the item: ‘As far as you can recall, has it happened over the previous 12 months that you have gone to work despite feeling that you really should have taken sick leave due to your state of health?’ with responses options 1 = no, never, 2 = yes, once, 3 = yes, 2 to 5 times, 4 = yes, more than 5 times (in this paper, the presenteeism variable was recoded into Yes/No format). Finally, we included an item relating to whether participants had accessed a centre (no; yes, once; yes, more than once; in the current analyses this has been recoded into Yes/No format).

### Statistical analysis

Data were analysed using IBM SPSS Version 26.0 [57]. To examine the relationship between wellbeing centre use and various constructs of interest, a series of moderation analyses was conducted. ANCOVA model or regression analyses were used, depending on the level of the dependent variable.

The following statistical analyses examining research questions (1–4) were performed: linear regression models exploring (1) associations between job-related factors and HCWs wellbeing, (2) the potential moderation between job stress and wellbeing centre use on HCWs wellbeing, (3) the potential moderation between job satisfaction and wellbeing centre use on HCWs wellbeing. An ANCOVA model examining the role of wellbeing centre use and presenteeism on HCWs wellbeing (4) was also performed, followed by (5) a binary logistic regression exploring factors (i.e., job stress, job satisfaction, wellbeing centre use and their interactions) potentially associated with turnover intentions among HCWs.

## Results

**Fig. 1:**
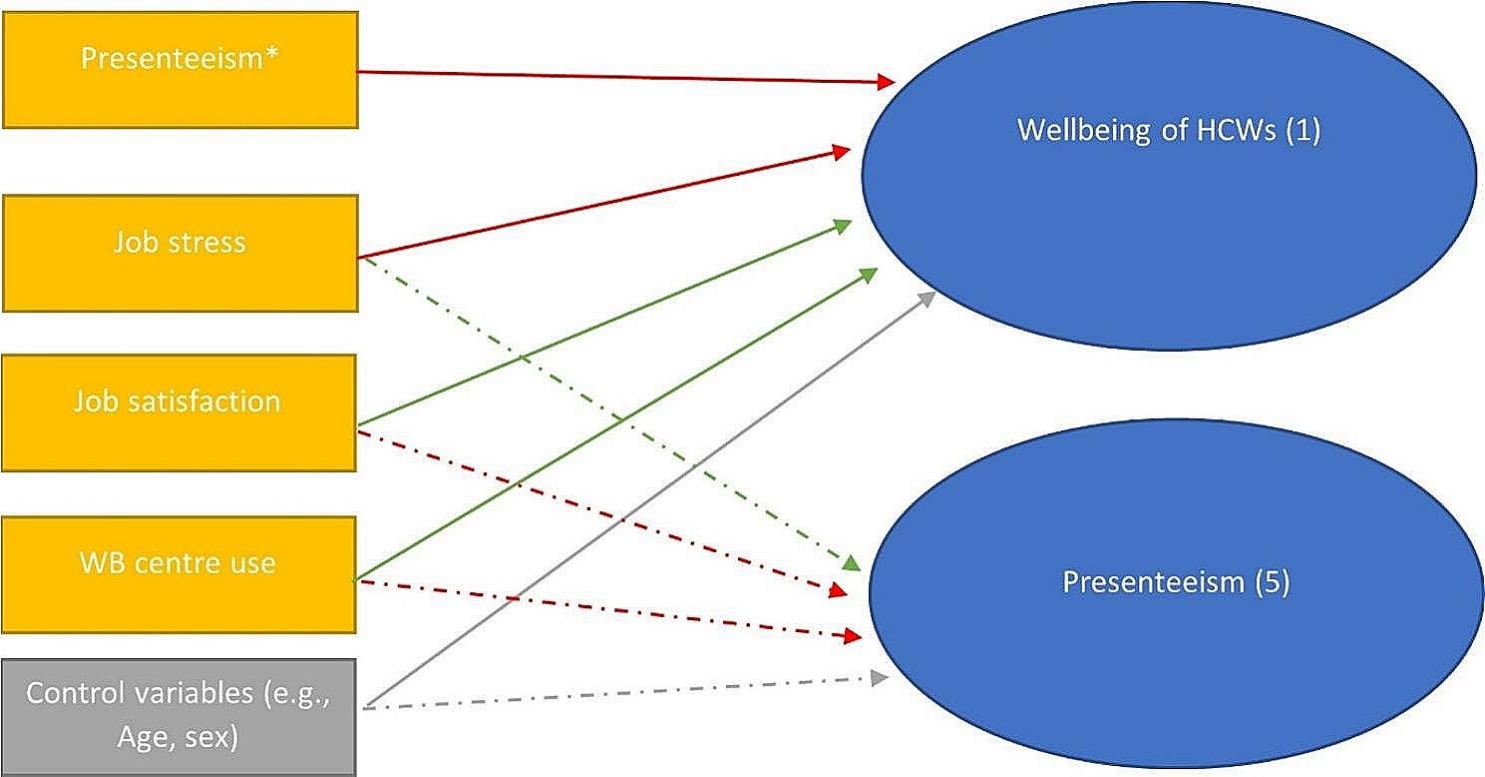
The associations tested in analysis 1 and 5

**Fig. 2:**
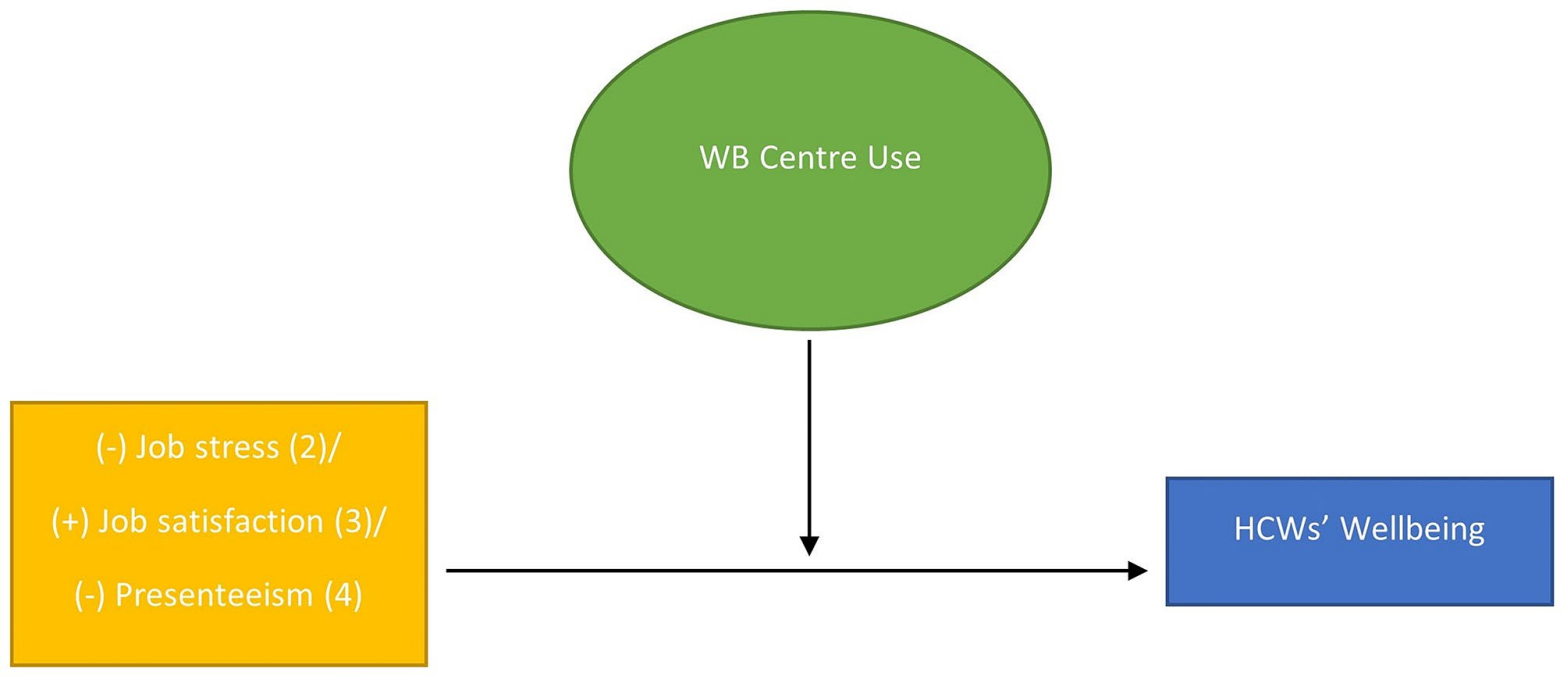
Moderation model tested in analysis 2–4

In this section, the results of the analyses explained above are presented. First, we demonstrate the associations between job-related factors and wellbeing (the first outcome of interest), followed by moderation analyses, and completed by an examination of factors linked to turnover intentions (the second outcome of interest). The more detailed information on statistical coefficients is provided in supplementary material. The models that were tested are presented graphically in Figs. 1 and 2.

*Conceptual models tested in this study: (Fig. 1: The associations tested in analysis 1 and 5). Note: Red arrows indicate a negative association between variables, green arrows indicate a positive association between variables, while grey arrows represent the role of control variables). (Fig. 2: Moderation model tested in analysis 2–4). Note: The +/- sign reflect the predicted direction of the relationships between the predictor variables and the outcome. The numbers in brackets represent the number of analysis where the relevant model was analysed.

*Although it is possible to examine presenteeism as a predictor of wellbeing, this was not the aim of this paper. In alignment with the focus of our paper (and previous literature: e.g., [18, 58–60]) we examined whether reporting presenteeism and accessing/not accessing a wellbeing centre might have affected HCWs wellbeing, rather than the reverse relationship.

### Analysis 1: job-related factors and wellbeing – an exploratory model

Of 819 respondents, data from 806 HCWs were used in the analysis (women: *n* = 721; men: *n* = 85). Age distribution was 16–20 years (1%, *n* = 6), 21–30 years (17.5%, *n* = 141), 31–40 years (22%, *n* = 176), 41–50 years (29%, *n* = 232), 51–60 years (27%, *n* = 219) and > 60 years (4%, *n* = 30). Two participants did not report their age.

To explore wellbeing among HCWs, all the examined predictor variables (i.e., wellbeing centre use, presenteeism, job satisfaction and job stress) were entered into a linear regression model (Table 2). Age and gender were used as control variables. The model explained 39% of variance in wellbeing scores (F(6,786) = 83.45, *p* < .001). Lower wellbeing was associated with not accessing the centres, higher job stress, lower job satisfaction, presenteeism, and younger age. There was no association with gender.

**Table 2:** Linear regression model predicting wellbeing of healthcare workers (*n* = 793)

Variable	B	*p* value	95% CI
Constant	3.23	< 0.001	2.96–3.49
Wellbeing centre use	0.08	< 0.001	0.05 – 0.12
Sex	− 0.09	0.15	− 0.21 – 0.03
Age	0.05	0.002	0.02 − 0.08
Job stress	− 0.17	< 0.001	− 0.21 – − 0.12
Job satisfaction	0.23	< 0.001	0.20 – 0.27
Presenteeism	− 0.15	< 0.001	− 0.19 – − 0.11

Since all predictor variables showed an effect on the wellbeing of HCWs, this warranted further exploration of a potential moderating effect of wellbeing centre use, in line with the aim of this study, which was examined in the subsequent analyses (2–4).

### Analysis 2: role of job stress and centre use for wellbeing scores

A linear regression model (*n* = 797) was used to determine whether centre use moderated the association between job stress and wellbeing (Table 3), with gender and age entered as control variables. The job stress variable was centred (i.e., subtracting the mean value from each data point for this variable; this is a preliminary step when examining interaction effects). The model explained 19% of variance in wellbeing (F(5,791) = 36.12, *p* < .001). Lower wellbeing was associated with higher job stress, and not accessing the centres, as well as younger age. There was no interaction between job stress and centre use on wellbeing scores, and no association with gender. This shows that accessing the wellbeing centres was positively associated with wellbeing, but this relationship did not differ according to the level of job stress.

**Table 3:** Linear regression model predicting wellbeing of HCWs, including the potential interaction between job stress and centre use (*n* = 797)

Variable	B	*p* value	95% CI
Constant	3.15	< 0.001	2.98–3.33
Wellbeing centre use	0.17	< 0.001	0.09 – 0.26
Sex	− 0.10	0.18	− 0.24 – 0.04
Age	0.06	0.002	0.02 − 0.10
Job stress	− 0.31	< 0.001	− 0.36 – − 0.26
Job stress x wellbeing centre use	− 0.01	0.83	− 0.05 – 0.04

### Analysis 3: role of job satisfaction and centre use for wellbeing scores

In a complementary fashion, a linear regression model (*n* = 798) was used to determine whether centre use (Yes/No) moderated the association between job satisfaction and wellbeing (Table 4), with gender and age entered as control variables. The job satisfaction variable was centred. The model explained 29% of variance in wellbeing (F(5,792) = 63.57, *p* < .001). Here, higher wellbeing was associated with higher job satisfaction, and accessing the centres, as well as older age. There was, however, no interaction between job stress and centre use on wellbeing scores, and no association with gender. This shows there was a positive association between accessing the wellbeing centres and wellbeing scores, but this relationship did not differ according to the level of job satisfaction.

**Table 4:** Linear regression model predicting wellbeing of healthcare staff, including the potential interaction between job satisfaction and centre use (*n* = 798)

Variable	B	*p* value	95% CI
Constant	3.14	< 0.001	2.97–3.30
Wellbeing centre use	0.15	< 0.001	0.07 – 0.23
Sex	− 0.04	0.60	− 0.17 – 0.10
Age	0.05	0.006	0.01 − 0.08
Job satisfaction	0.30	< 0.001	0.27 – 0.34
Job satisfaction x wellbeing centre use	0.02	0.24	− 0.01 – 0.06

### Analysis 4: relationship between presenteeism, wellbeing and centre use

We examined whether the well-known relationship between presenteeism and wellbeing is moderated by centre use. A 2 × 2 ANCOVA was run, presenteeism (coded as Yes: *n* = 557, No: *n* = 255) and centre use (coded as Yes: *n* = 447, No: *n* = 365) were entered as independent factors, with wellbeing level constituting a dependent variable. Age and gender were included as covariates (gender showed no effect: *p* = .35, whereas age showed a significant effect: *p* = .007). Results showed a significant main effect of presenteeism (*p* < .001), as well as centre use (*p* = .026).

There was a significant interaction effect between presenteeism and centre use (*p* = .008) (see Figure S1 in supplementary material). Simple main effects analysis revealed a significant difference in wellbeing in relation to presenteeism (*p* < .001). Those reporting presenteeism and who accessed the centre (M = 3.30, SE = 0.04) had higher wellbeing than those who accessed the centre but did not report presenteeism (M = 3.06, SE = 0.04). There was no difference in wellbeing scores (accessed centres: M = 3.59, SE = 0.06; did not access centres: M = 3.61, SE = 0.06) for those in the ‘no presenteeism’ group, irrespective of whether or not they accessed the centres (*p* = .81). Wellbeing scores differed, however, among those who accessed the centres (*p* < .001) and was higher for those with no presenteeism (M = 3.59, SE = 0.06), and lower for those reporting presenteeism (M = 3.30, SE = 0.04). The same was true for those who did not access the centres (*p* < .001), with higher wellbeing scores (M = 3.61, SE = 0.06) among the no presenteeism group, and lower wellbeing scores (M = 3.06, SE = 0.04) among the presenteeism group.

While analysis 1 showed that presenteeism was associated with low wellbeing, analysis 4 shows that this relationship is moderated by centre use. HCWs reporting presenteeism that *had not* accessed the centres had significantly lower wellbeing than those with presenteeism that *had* accessed the centres.

### Analysis 5: factors associated with turnover intentions

Finally, a model predicting turnover intentions (the second outcome of interest) was performed. Participants were grouped into those who indicated considering leaving their job (*n* = 246, 31.1%), and their counterparts (*n* = 544, 68.9%). Following on from the previous models, a moderating role of wellbeing centre use on job stress and job satisfaction was tested, with age and gender as control variables. A binary logistic regression model was run. The overall model was significant (Χ^2^ = 224.64, *p* < .001), explained 35% of the variance (Nagelkerke R^2^ = 0.35), and correctly classified 78.5% of cases. As shown in Table 5, centre use was not significantly associated with turnover intentions, and did not significantly interact with job stress or job satisfaction. Job stress and job satisfaction were the only significant factors in this model. This shows that HCWs were more likely to consider leaving their jobs when their job stress was high, and job satisfaction low. There were no significant associations with age or gender.

**Table 5:** Binary logistic regression model predicting turnover intentions of HCWs, including the potential interaction between job stress and wellbeing centre use (*n* = 797)

Variable	Odds ratio	*p* value	95% CI
Constant	2.45	< 0.001	-
Wellbeing centre use	0.74	0.13	0.50–1.10
Sex	1.36	0.33	0.74–2.48
Age	1.11	0.19	0.95–1.29
Job stress	0.62	< 0.001	0.50 – 0.78
Job stress x wellbeing centre use	0.83	0.09	0.66–1.03
Job satisfaction	2.79	< 0.001	2.31–3.36
Job satisfaction x wellbeing centre use	0.92	0.39	0.76–1.11

## Discussion

The main aim of this study was to examine the relationship between accessing a supported wellbeing centre and HCWs wellbeing, during the first wave of the COVID-19 pandemic in the UK. These centres comprised access to a high-quality rest space and peer-to-peer psychological first aid (hence ‘supported’); they were rapidly mobilised within weeks of COVID-19 being declared a pandemic, and were globally, the first wellbeing interventions of their kind [1, 29]. This secondary analysis of COVID-Well data [29] shows that accessing a supported wellbeing centre was independently, and positively associated with wellbeing in HCWs. This demonstrates a clear benefit to the healthcare workforce, a population in which low wellbeing was evident before [61] and during the pandemic [5]. Our findings build on two prior COVID-Well studies showing that (a) the wellbeing centres were highly accessed by HCWs [29] and, (b) that the existence of centres as high-quality break spaces, together with the provision of peer-to-peer psychological first aid, was valued by the workforce [1]. Nonetheless, further research is needed to establish the effectiveness of psychological first aid for HCWs on wellbeing outcomes, the evidence for which, at the time of study, was defined as low certainty [26].

The COVID-Well studies reported on the first evaluation of the implementation of supported wellbeing centres in healthcare settings, conducted at the outset of the pandemic in 2020, and demonstrated that this provision played an important role in workforce wellbeing [1, 29]. Subsequently, in February 2021, the UK Government funded 40 national ‘NHS staff mental health and wellbeing hubs’ which gave health and social care workers access to mental health support that was provided by dedicated local mental health services. Recent evaluations of these wellbeing or ‘resilience hubs’ further demonstrate the need for psychological support for those working in health and social care services, and the value of such interventions for supporting wellbeing during the pandemic [62]. Due to governmental funding being stopped these NHS staff mental health and wellbeing hubs closed in March 2023 [63], although there have been numerous calls to reinstate the funding needed (e.g., from Royal College of Nursing, British Psychological Society, Royal College of Psychiatrists, and others: e.g. [64]) since the importance of ongoing psychological support for the health and care workforce is well recognised. The findings of our study, and others (e.g. [1, 29, 62]) highlight the benefits of interventions to support HCW wellbeing (e.g., through provision of supported wellbeing centres, or resilience hubs). Our prior qualitative interviews with HCWs who had accessed our wellbeing centres suggests that the perceived value of such centres expands beyond the context of emergencies [1]. Within the first COVID-Well study, we provided the first published data on costs associated with the set-up and delivery of COVID-19 workforce wellbeing centres [29]. A full economic analysis would be required to establish the cost-benefit of wellbeing centres, although this exploratory cost data was particularly timely to inform other healthcare organisations of the process and resources required to establish similar centres. The set-up and implementation of wellbeing centres, especially in budget-constrained environments, can be challenging. Nonetheless, the COVID-Well studies lead us to anticipate (similarly to others [e.g., 40, 65]) that providing HCWs with access to a wellbeing centre or rest space (e.g., a small, dedicated space, with access to refreshments, calming environment, where one can share their work experiences and support with other colleagues, or listen to relaxation scripts, or decompress etc.), even if resource limited, could to at least some basic degree support the wellbeing of HCWs in their place of work. Improving the wellbeing of HCWs may potentially facilitate better patient care, in ‘normal’ times, as well as times of crisis. In alignment with others (e.g., [66]), we reiterate that the need for wellbeing hubs or centres for HCWs, especially combined with access to professional psychological support, has never seemed greater and that a long-term investment in these initiatives is clearly needed.

When exploring predictors of wellbeing, we corroborated previous evidence showing a negative relationship between job stress and wellbeing [15, 16], a negative relationship between presenteeism and wellbeing [17–19], and a positive relationship between job satisfaction and wellbeing in HCWs [20–22]. Wellbeing was lower in younger workers - this aligns with other research showing lower wellbeing and/or higher prevalence of adverse mental health outcomes in younger HCWs [9, 67–73]. Similar age-related patterns have been observed in general population samples [74, 75]. This disproportionate impact of the pandemic on mental wellbeing of younger workers could reflect caregiving responsibilities for many (e.g., managing childcare around work and social restrictions and associated fear of disease transmission), shorter time in their job role, less experience of coping with difficult, complex, or life-threatening situations, concerns relating to fewer work or education opportunities, job insecurity, and financial insecurity from lower income [76].

Job stress was prevalent in HCWs, before [77] and during [5, 29, 78] the COVID-19 pandemic, and has implications for individual health and wellbeing, effectiveness of healthcare organisations and care quality [79]. This has been observed globally; during the first wave of the pandemic, Couarraze and colleagues [80] described stress in HCWs across occupational groups and geographical regions (*n* = 13,537, 44 countries). Pre-pandemic, interventions targeting stress were found to have positive outcomes for nurses’ health and/or wellbeing [24]. During the pandemic, a review highlighted the paucity and heterogeneity of organisational psychological support intervention protocols for HCWs aimed at mitigating the impact of occupational stressors associated with COVID-19 [81]. Emerging individual-level interventions to mitigate stress and the mental health impacts of COVID-19 include an e-support package, psychoeducation, mental health promotion, mindfulness and talking therapies [27, 82–85]. Here, we did not identify any moderating effect of wellbeing centre access on the relationship between job stressfulness and wellbeing, despite qualitative research showing stress reduction and positive impacts on wellbeing through enabling opportunities to take work breaks and having access to social and psychological support within the centres [1]. Research conducted prior to the pandemic also suggested that rest breaks and the quality of break areas benefit HCWs (and the patients they serve) [86]. The lack of moderating effect here could potentially be explained by the use of a single-item measure of job stressfulness which may not have picked up on specific, acute stressors and complex relationships between them, that may influence the stress/wellbeing relationship in the context of a crisis (e.g., escalating global pandemic context, uncertainty and lack of job control, problems with access to personal protective equipment (PPE), rapidly changing roles, excessive workload, etc.). Alternatively, it may reflect the value of wellbeing centres in improving wellbeing, albeit alongside a certain level of unmodifiable stress that is naturally present in healthcare professional’s job roles, particularly during crisis situations, such as a pandemic.

Presenteeism is high in healthcare workers, higher than pre-pandemic levels [87], and is known to increase with job stress [88, 89]. In the sample from which our data are drawn, 68% of respondent reported presenteeism during the first surge of COVID-19 [29], and higher rates have been observed in HCWs elsewhere (e.g., 82%, USA) [90]. Presenteeism carries a high economic burden due to negative impacts on productivity [91, 92] and in healthcare, it has been described as a ‘public health hazard’ due to risk of infectious disease transmission in vulnerable patient populations [93]. In our study, wellbeing centre use moderated the link between presenteeism and wellbeing. That is, HCWs who reported presenteeism and had not accessed the centres showed a significantly lower level of wellbeing than those reporting presenteeism but who accessed the wellbeing centres. This suggests that for those who were present at work despite feeling unwell, accessing the wellbeing centres appeared to have a protective influence on wellbeing – perhaps providing greater respite and restoration for those who were not in optimal health. Future research might explore what motivated some, but not all, of the HCWs that reported presenteeism to use the wellbeing centres. This may be related to known barriers to service access, such as proximity of work areas to the centres, promotion of centres to all occupational groups, managerial and team support for wellbeing, and the challenges surrounding taking work breaks alongside requirements for donning and doffing PPE [1].

Job satisfaction is important in healthcare professions since it is associated with work absenteeism [94], intentions to leave and turnover [95]. Implementing strategies to enhance job satisfaction are therefore of value and this aligns with the 2019 recommendations provided by the National Academies of Sciences, Engineering, and Medicine Studies [[96]: Recommendation 1B] which advocate for the prioritisation of interventions that have potential to promote clinicians sense of meaning in life and at work. Our analysis confirms that accessing a wellbeing centre did not moderate the relationship between job satisfaction and wellbeing or influence turnover intentions. This is not unexpected since the centres were aimed at improving wellbeing (which was achieved), rather than job satisfaction or turnover intention, per se. Nonetheless, these variables are related, since low job satisfaction predicts turnover intention [97], particularly when wellbeing is low [98]. Almost one third of our sample reported intention to leave their job [29] which is broadly comparable to other studies with healthcare workers (e.g., 31.7%: [99]; 27.7%: [100]). Fear of COVID-19 has exacerbated turnover intentions in frontline HCWs [101]. The unexplained variance in our model of predictors of turnover intention, however, suggests that other factors may be salient here at individual level (e.g., emotional exhaustion, depression, job stress, fatigue, emotional labour, work engagement, job satisfaction, professional self-concept), unit level (e.g., work conditions, interpersonal relationships, and unit culture), and organisational level (e.g., organizational commitment, person − organization fit, job embeddedness, organizational justice, organizational socialization and internal marketing of the organization) [49]. Alternatively, intention to leave may simply reflect natural processes in people’s career pathways, such as anticipation of retirement or professional development into another job role.

The association observed between job satisfaction and wellbeing (as well as turnover intentions), irrespective of wellbeing centre use, supports the need for strategies to enhance job satisfaction in HCWs. Many approaches have shown promise; studies have accentuated the influence of empowerment and transformational leadership [102] and emotional competence [103, 104] on job satisfaction among HCWs. Participation in ‘Compassion Rounds’ has shown to increase job satisfaction, by fostering emotional expression, teamwork, and communication [105]. Job satisfaction has also increased following structured ‘huddles’ and peer recognition schemes for HCWs [106] and yoga practice for nurse academicians [107]. A systematic review and meta-analysis of interventions developed to increase job satisfaction in nurses found that interventions were primarily educational and consisted of workshops, educational sessions, lessons, and training sessions [108]. Notably, this review showed that organisational strategies to foster the *intrinsic* motivation of employees (e.g., spiritual intelligence, professional identity, and awareness) were more effective in increasing job satisfaction than *extrinsic* factors (e.g., salary and rewards) [108], a finding echoed in earlier studies [109].

**Fig. 3:**
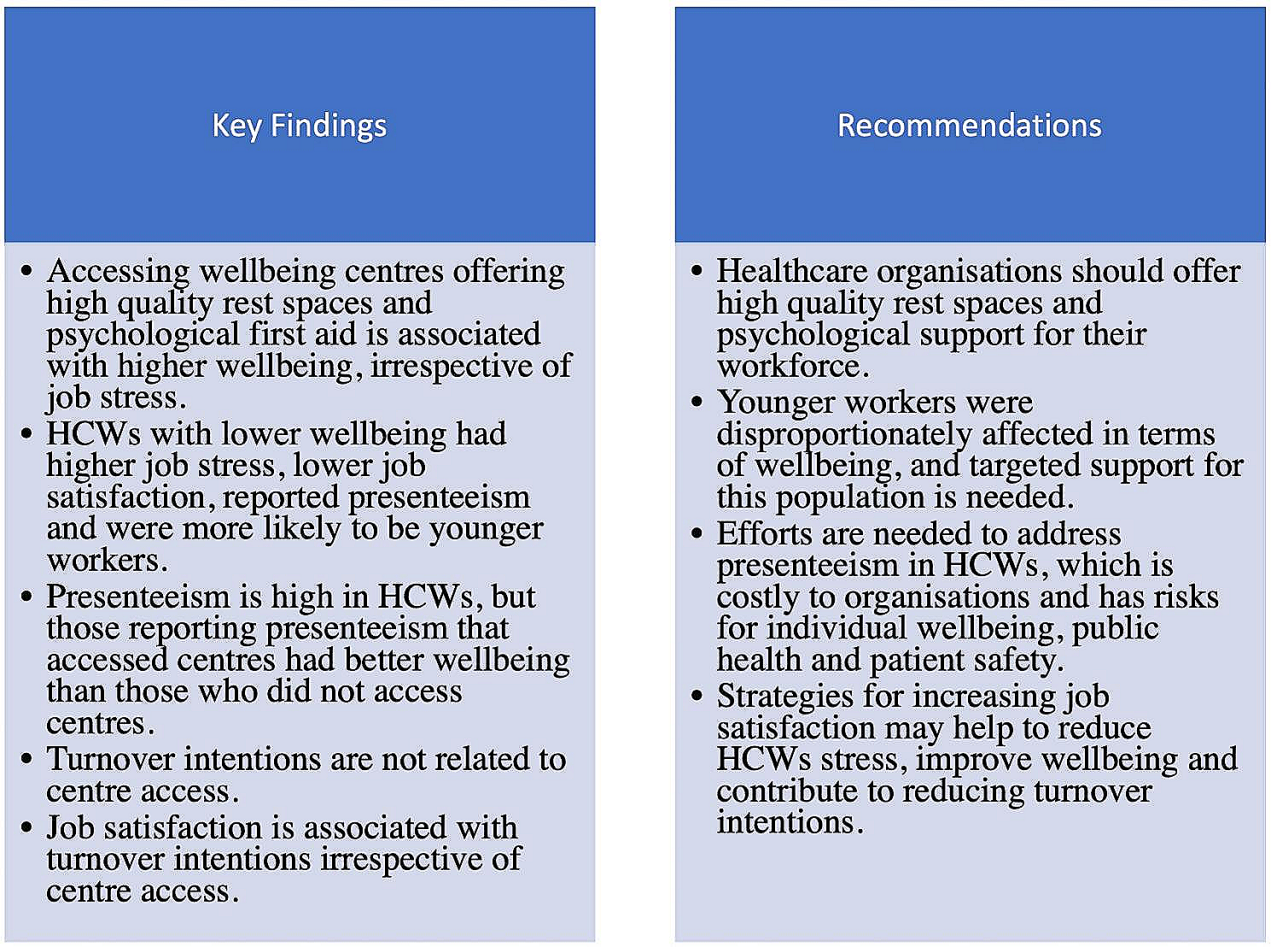
Key findings and recommendations

This study provides insights into the factors associated with wellbeing in HCWs during the first surge of the COVID-19 pandemic in the UK. We provide insights into the value of supported wellbeing centres as one approach taken in an acute hospital setting, to mitigating the impact of a pandemic on the psychological wellbeing of HCWs. While there were demonstrable benefits to this approach, it should be recognised that wellbeing support requires intervention at individual, unit-, and organisational-level. In the UK, whole-system approaches to improving the health and wellbeing of healthcare workers have been advocated [110]. This refers to approaches that include identification and response to local need, engagement of the whole workforce (staff at all levels), and the involvement, visible leadership from, and up-skilling of, management and board-level staff. COVID-19 exacerbated challenges that already existed for healthcare workers. Therefore, strategies and interventions that showed benefit for workforce wellbeing during the pandemic should extend beyond times of crisis and be available in the long-term. Key findings and recommendations are shown in Fig. 3.

### Study limitations

Data were collected from a self-selected sample of employees at a single NHS Trust in England, albeit survey participants could have been based on any of this Trust’s three hospital sites, accessing wellbeing centres available at two of those sites. Data collection took place during the first wave of COVID-19, and while timely, this was an uncertain and rapidly changing local and national context. Since the survey was administered during the pandemic, it was kept purposely brief to maximise response. Therefore, there is a risk of unmeasured confounding since only age and gender were collected and included as covariates in our analytic models and no other sociodemographic data were available (e.g., marital status, living arrangements, caregiving roles, etc.). To maximise survey completion rate during an exceptionally busy and challenging period for HCWs, we used single-item measures of job stressfulness, job satisfaction, presenteeism and turnover intentions.

The cross-sectional study design reduces the ability to determine causality or analyse changes in variables (e.g., wellbeing, or centre access) over time. Longitudinal data would provide further insight into the predictive value of wellbeing centres for individual and organisational outcomes. However, a group comparison between those who did versus those who not access wellbeing centres lends some support (albeit caveated by the risk of unobserved differences between the groups) for their protective role, although this needs to be explored further in a longitudinal design. Findings may not be directly generalisable to other geographical regions, or at a different time but likely have relevance beyond the context of a pandemic.

## Conclusion

Accessing wellbeing centres was associated with higher wellbeing of HCWs, irrespective of job stress. HCWs with lower wellbeing had higher job stress, lower job satisfaction, reported presenteeism and were more likely to be younger workers. The relationship between presenteeism and wellbeing was moderated by centre access; those reporting presenteeism that accessed centres had better wellbeing than those who did not access centres. Job satisfaction was related to turnover intentions irrespective of centre access. We advocate that healthcare organisations should provide high-quality rest spaces and psychological support for HCWs. This should be part of a whole-system approach to improving the health and wellbeing of healthcare workers. There is a need for strategies and interventions aimed at enhancing job satisfaction and reducing presenteeism which could contribute to reducing turnover intentions and may ultimately impact on individuals, organisations, and care quality. Targeted wellbeing support is needed for younger workers for whom wellbeing was disproportionately affected during the pandemic.

### Electronic supplementary material

Below is the link to the electronic supplementary material.

Supplementary Material 1

## Data Availability

The datasets used and/or analysed during the current study are available from the corresponding author on reasonable request.

## Abbreviations

ANCOVA: Analysis of covariance
CIs: Confidence intervals
COVID-19: Coronavirus
HCWs: Healthcare workers
NHS: National Health Service
PFA: Psychological first aid
SPSS: Statistical Package for the Social Sciences
TIDieR: Template for intervention description and replication
UK: United Kingdom
USA: United States of America
