# Supplementary material for: Workforce wellbeing centres and their positive role for wellbeing and presenteeism in healthcare workers during the COVID-19 pandemic: secondary analysis of COVID-Well data
Source: BMC Health Serv Res. 2024 Mar 6;24:302. doi: 10.1186/s12913-024-10730-9 (PMC10918935; doi:10.1186/s12913-024-10730-9)
Supplement: Supplementary file 1 — Supplementary Material 1 [file 12913_2024_10730_MOESM1_ESM.docx]

**Supplementary file.** Additional detail for study results.

Wellbeing centre use

No

Yes

Wellbeing level

Presenteeism

Yes No


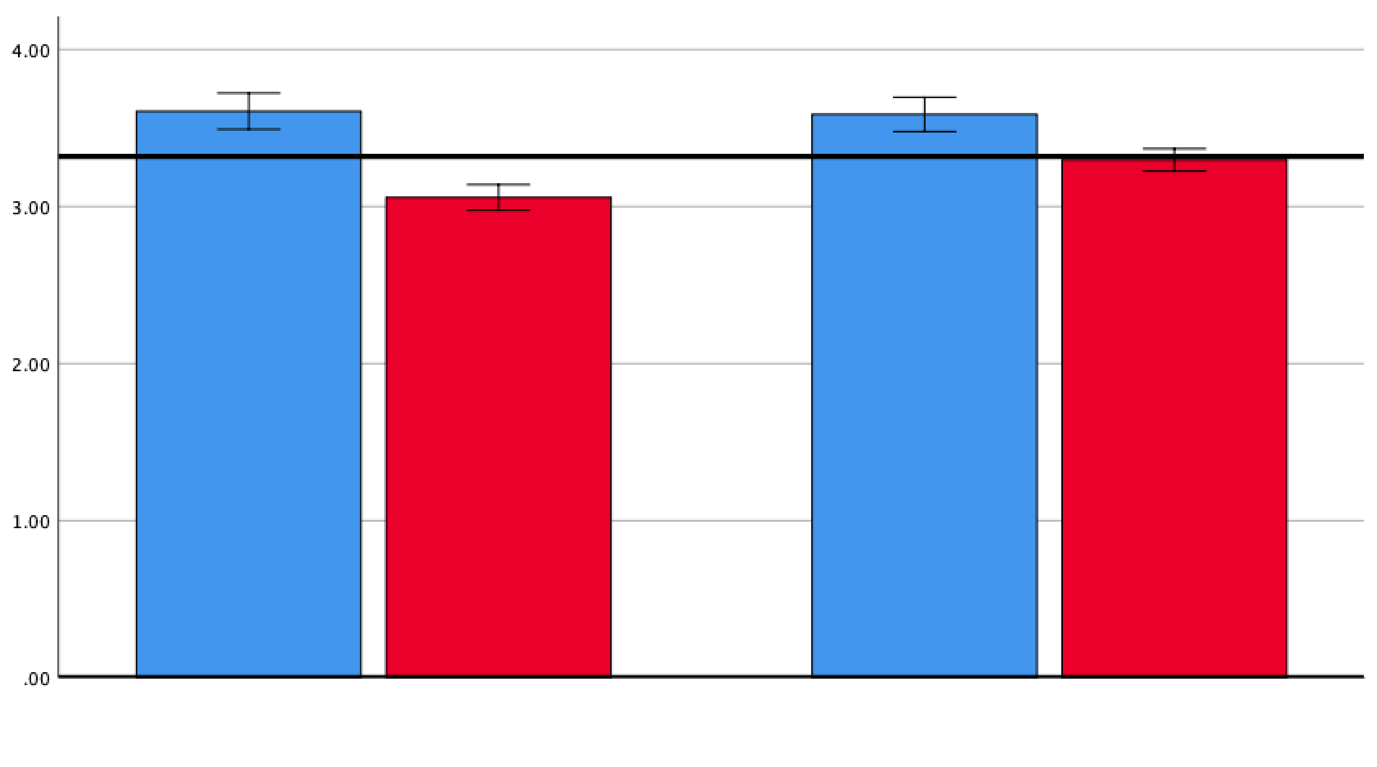


Figure S1. Wellbeing scores in relation to presenteeism and centre use.

Note: Error bars represent the 95% CIs.

Supplementary text

Below are the descriptions of the results (analyses 1-5 from the main manuscript) and tables with more detailed statistical information provided, which was omitted from the main manuscript to help with the text flow.

Analysis 1

To explore wellbeing among HCWs, all the examined predictor variables (i.e., wellbeing centre use, presenteeism, job satisfaction and job stress) were entered into a linear regression model (Table S1). Age and gender were used as control variables. The model explained 39% of variance in wellbeing scores (F(6,786) = 83.45, p < .001). Lower wellbeing was predicted by not accessing the centres (β = .12, p < .001), higher job stress (β = -.22, p < .001), lower job satisfaction (β = .39, p < .001), presenteeism (β = -.22, p < .001), and younger age (β = .09, p =.002). There was no effect of gender (β = -.04, p =.15).

Table S1. Linear regression model predicting wellbeing of healthcare workers (n=793).

| Variable | B | SE | β | p value | 95% CI |
| --- | --- | --- | --- | --- | --- |
| Constant | 3.23 | .14 | - | <.001 | 2.96 – 3.49 |
| Wellbeing centre use | .08 | .02 | .12 | <.001 | .05 – .12 |
| Sex | -.09 | .06 | -.04 | .15 | -.21 – .03 |
| Age | .05 | .02 | .09 | .002 | .02 - .08 |
| Job stress | -.17 | .02 | -.22 | <.001 | -.21 – -.12 |
| Job satisfaction | .23 | .02 | .39 | <.001 | .20 – .27 |
| Presenteeism | -.15 | .02 | -.22 | <.001 | -.19 – -.11 |

Analysis 2

A linear regression model (n=797) was used to determine whether centre use moderated the effect of job stress on wellbeing (Table S2), with gender and age entered as control variables. The job stress variable was centred (i.e., subtracting the mean value from each data point for this variable; this is a preliminary step when examining interaction effects). The model explained 19% of variance in wellbeing (F(5,791) = 36.12, p < .001). Lower wellbeing was predicted by higher job stress (β = -.41, p < .001), and not accessing the centres (β = .13, p < .001), as well as younger age (β = .10, p =.002). There was no interaction between job stress and centre use on wellbeing scores (β = -.01, p = .83), and no effect of gender (β = -.04, p = .18). This shows that accessing the wellbeing centres had a positive effect on wellbeing, but this effect did not differ according to the level of job stress.

Table S2. Linear regression model predicting wellbeing of HCWs, including the potential interaction between job stress and centre use (n=797).

| Variable | B | SE | β | p value | 95% CI |
| --- | --- | --- | --- | --- | --- |
| Constant | 3.15 | .09 | - | <.001 | 2.98 – 3.33 |
| Wellbeing centre use | .17 | .04 | .13 | <.001 | .09 – .26 |
| Sex | -.10 | .07 | -.04 | .18 | -.24 – .04 |
| Age | .06 | .02 | .10 | .002 | .02 - .10 |
| Job stress | -.31 | .03 | -.41 | <.001 | -.36 – -.26 |
| Job stress x Wellbeing centre use | -.01 | .03 | -.01 | .83 | -.05 – .04 |

Analysis 3

In a complementary fashion, a linear regression model (n=798) was used to determine whether centre use (Yes/No) moderated the effect of job satisfaction on wellbeing (Table S3), with gender and age entered as control variables. The job satisfaction variable was centred. The model explained 29% of variance in wellbeing (F(5,792) = 63.57, p < .001). Here, higher wellbeing was predicted by higher job satisfaction (β = .51, p < .001), and accessing the centres (β = .11, p < .001), as well as older age (β = .08, p = .006). There was, however, no interaction between job stress and centre use on wellbeing scores (β = .04, p = .24), and no effect of gender (β = -.02, p = .60). This shows there was a positive effect of accessing the wellbeing centres on wellbeing scores, but this effect did not differ according to the level of job satisfaction.

Table S3. Linear regression model predicting wellbeing of healthcare staff, including the potential interaction between job satisfaction and centre use (n=798).

| Variable | B | SE | β | p value | 95% CI |
| --- | --- | --- | --- | --- | --- |
| Constant | 3.14 | .08 | - | <.001 | 2.97 – 3.30 |
| Wellbeing centre use | .15 | .04 | .11 | <.001 | .07 – .23 |
| Sex | -.04 | .07 | -.02 | .60 | -.17 – .10 |
| Age | .05 | .02 | .08 | .006 | .01 - .08 |
| Job satisfaction | .30 | .02 | .51 | <.001 | .27 – .34 |
| Job satisfaction x WB centre use | .02 | .02 | .04 | .24 | -.01 – .06 |

Analysis 4

We examined whether the well-known relationship between presenteeism and wellbeing is moderated by centre use. A 2x2 ANCOVA was run, presenteeism (coded as Yes: n=557, No: n=255) and centre use (coded as Yes: n=447, No: n=365) were entered as independent factors, with wellbeing level constituting a dependent variable. Age and gender were included as covariates (gender showed no effect: *F*(1,791) = .86, *p* = .35, partial η^2^ = .001, whereas age showed a significant effect: *F*(1,791) = 7.25, *p* = .007, partial η^2^ = .009). Results showed a significant main effect of presenteeism (*F*(1,791) = 73.58, *p* < .001, partial η^2^ = .09), as well as centre use (*F*(1,791) = 4.97, *p* = .026, partial η^2^ = .01).

There was a significant interaction effect between presenteeism and centre use (*F*(1,791) = 7.04, *p* = .008, partial η^2^ = .01) (Fig. 2). Simple main effects analysis revealed a significant difference in wellbeing in relation to presenteeism (*F*(1, 791) = 18.65, *p* < .001, η_p_^2^ = .02). Those reporting presenteeism and who accessed the centre (M=3.30, SE=.04) had higher wellbeing than those who accessed the centre but did not report presenteeism (M=3.06, SE=.04). There was no difference in wellbeing scores (accessed centres: M=3.59, SE=.06; did not access centres: M=3.61, SE=.06) for those in the ‘no presenteeism’ group, irrespective of whether or not they accessed the centres (*F*(1, 791) = .06, *p* = .81, η_p_^2^ < .001). Wellbeing scores differed, however, among those who accessed the centres (*F*(1, 791) = 19.05, *p* < .001, η_p_^2^ = .02) and was higher for those with no presenteeism (M=3.59, SE=.06), and lower for those reporting presenteeism (M=3.30, SE=.04). The same was true for those who did not access the centres (*F*(1, 791) = 58.48, *p* < .001, η_p_^2^ = .07), with higher wellbeing scores (M=3.61, SE=.06) among the no presenteeism group, and lower wellbeing scores (M=3.06, SE=.04) among the presenteeism group.

While analysis 1 showed that presenteeism leads to low wellbeing, analysis 4 shows that this relationship is moderated by centre use. HCWs reporting presenteeism that *had not* accessed the centres had significantly lower wellbeing than those with presenteeism that *had* accessed the centres (see Figure S1).

Analysis 5

Finally, a model predicting turnover intentions (the second outcome of interest) was performed. Participants were grouped into those who indicated considering leaving their job (n=246, 31.1%), and their counterparts (n=544, 68.9%). Following on from the previous models, a moderating role of wellbeing centre use on job stress and job satisfaction was tested, with age and gender as control variables. A binary logistic regression model was run. The overall model was significant (Χ^2^ = 224.64, p < .001), explained 35% of the variance (Nagelkerke R^2^ = .35), and correctly classified 78.5% of cases. As shown in Table S4, centre use was not a significant predictor of turnover intentions (B = -.30, p = .13; Wald = 2.26; odds = .74) and did not significantly interact with job stress (B = -.19, p = .09; Wald = 2.88; odds = .83) or job satisfaction (B = -.08, p = .39; Wald = .73; odds = .92). Job stress and job satisfaction were the only significant factors in this model (job stress: B = -.48, p < .001; Wald = 17.86; odds = .62; job satisfaction: B = 1.03, p < .001; Wald = 115.55; odds = 2.79). There was no significant effect of age or gender (ps > .05).

Table S4. Binary logistic regression model predicting turnover intentions of HCWs, including the potential interaction between job stress and wellbeing centre use (n=797).

| Variable | B | SE | Wald | p value | Odds ratio | 95% CI |
| --- | --- | --- | --- | --- | --- | --- |
| Constant | .90 | .24 | 13.99 | <.001 | 2.45 | - |
| Wellbeing centre use | -.30 | .20 | 2.26 | .13 | .74 | .50 – 1.10 |
| Sex | .30 | .31 | .97 | .33 | 1.36 | .74 – 2.48 |
| Age | .10 | .08 | 1.73 | .19 | 1.11 | .95 - 1.29 |
| Job stress | -.48 | .11 | 17.86 | <.001 | .62 | .50 – .78 |
| Job stress x wellbeing centre use | -.19 | .11 | 2.88 | .09 | .83 | .66 – 1.03 |
| Job satisfaction | 1.03 | .10 | 115.55 | <.001 | 2.79 | 2.31 – 3.36 |
| Job satisfaction x wellbeing centre use | -.08 | .10 | .73 | .39 | .92 | .76 – 1.11 |
